# Expression and Function of GABA Receptors in Myelinating Cells

**DOI:** 10.3389/fncel.2020.00256

**Published:** 2020-08-21

**Authors:** Mari Paz Serrano-Regal, Laura Bayón-Cordero, Rainald Pablo Ordaz, Edith Garay, Agenor Limon, Rogelio O. Arellano, Carlos Matute, María Victoria Sánchez-Gómez

**Affiliations:** ^1^Laboratory of Neurobiology, Achucarro Basque Center for Neuroscience, Leioa, Spain; ^2^Department of Neurosciences, University of the Basque Country (UPV/EHU), Leioa, Spain; ^3^Centro de Investigación Biomédica en Red de Enfermedades Neurodegenerativas (CIBERNED), Leioa, Spain; ^4^Laboratorio de Neurofisiología Celular, Instituto de Neurobiología, Universidad Nacional Autónoma de México, Juriquilla, Mexico; ^5^Department of Neurology, Mitchell Center for Neurodegenerative Diseases, University of Texas Medical Branch, Galveston, TX, United States

**Keywords:** GABA, GABA receptor, oligodendrocyte, Schwann cell, differentiation, myelination

## Abstract

Myelin facilitates the fast transmission of nerve impulses and provides metabolic support to axons. Differentiation of oligodendrocyte progenitor cells (OPCs) and Schwann cell (SC) precursors is critical for myelination during development and myelin repair in demyelinating disorders. Myelination is tightly controlled by neuron-glia communication and requires the participation of a wide repertoire of signals, including neurotransmitters such as glutamate, ATP, adenosine, or γ-aminobutyric acid (GABA). GABA is the main inhibitory neurotransmitter in the central nervous system (CNS) and it is also present in the peripheral nervous system (PNS). The composition and function of GABA receptors (GABARs) are well studied in neurons, while their nature and role in glial cells are still incipient. Recent studies demonstrate that GABA-mediated signaling mechanisms play relevant roles in OPC and SC precursor development and function, and stand out the implication of GABARs in oligodendrocyte (OL) and SC maturation and myelination. In this review, we highlight the evidence supporting the novel role of GABA with an emphasis on the molecular identity of the receptors expressed in these glial cells and the possible signaling pathways involved in their actions. GABAergic signaling in myelinating cells may have potential implications for developing novel reparative therapies in demyelinating diseases.

## Introduction

Glial cells express a vast repertoire of receptors and transporters for neurotransmitters and neuromodulators and respond to axonal signals, being key and active elements of the nervous system (Allen and Lyons, 2018). In vertebrates, oligodendrocytes (OLs) and Schwann cells (SCs) are the myelin-forming glia of the central nervous system (CNS) and peripheral nervous system (PNS), respectively. These cells are responsible for myelin building and maintenance, a function highly regulated by neuronal activity (Gibson et al., 2014; Mitew et al., 2018). Myelin speeds up nerve impulse propagation and provides metabolic and trophic support to axons (Nave and Trapp, 2008; Kidd et al., 2013; Philips and Rothstein, 2017). Thus, myelination represents the major function of these cells, although they carry it out with some differences; while OLs can myelinate multiple axons simultaneously, each SC wraps one single axon (Jessen and Mirsky, 2005; Nave and Trapp, 2008). Regarding their specific characteristics, oligodendroglial cells represent a highly diverse and specialized cell population (Marques et al., 2016). Mature myelinating OLs develop from glial precursors named oligodendrocyte progenitor cells (OPCs), which constitute the main proliferating cell type in the adult CNS (Dawson et al., 2003). On the other hand, SCs derive from SC precursors, which differentiate into immature SCs. These immature SCs can generate both myelinating and non-myelinating SCs (or Remak glia) according to PNS requirements, like the presence of specific signals in the microenvironment and the diameter of axons in their vicinity (Jessen and Mirsky, 2005, 2019; Kidd et al., 2013).

Differentiation of OPCs and SC precursors is necessary for remyelination in demyelinating diseases like multiple sclerosis (MS) and myelin formation in dysmyelinating diseases such as leukodystrophies in the CNS or Charcot-Marie Tooth in the PNS. In this regard, understanding the mechanisms of action involved in this complex neuron-glia crosstalk will help us in the search for new therapeutic approaches in these pathologies.

Neuronal activity and several signals such as transcriptional and growth factors, axonal ligands, hormones, extracellular matrix components or neurotransmitters regulate OPC/SC precursor differentiation and myelination. Among them, GABAergic signaling has attracted great interest in the last years (Procacci et al., 2013; Zonouzi et al., 2015; Arellano et al., 2016; Hamilton et al., 2017; Serrano-Regal et al., 2020).

GABA, which is present both in the CNS and PNS, exerts an excitatory role during development to modulate neuronal growth and synapse formation (Ben-Ari, 2002). It acts mostly through ionotropic GABA_A_ (GABA_A_Rs) and metabotropic GABA_B_ receptors (GABA_B_Rs) that are well described in neurons but not yet fully characterized in myelinating cells. Although the expression of GABA receptors (GABARs) in OL/SC precursor lineages is widely documented (von Blankenfeld et al., 1991; Williamson et al., 1998; Magnaghi et al., 2004; Luyt et al., 2007; Arellano et al., 2016; Serrano-Regal et al., 2020), their role in differentiation and myelination is a matter of ongoing research.

In this review, we recapitulate recent evidence about GABA_A_ and GABA_B_ receptor expression and function in oligodendroglial and SCs, together with the implication of the GABAergic signaling in OPC/SC differentiation and myelination. Furthermore, we discuss possible signaling pathways involved in these events and their relevance to develop new therapies to treat demyelinating and dysmyelinating diseases.

## Expression of GABARs in Oligodendroglial and Schwann Cells

### GABA_A_ Receptors

**Figure 1:**
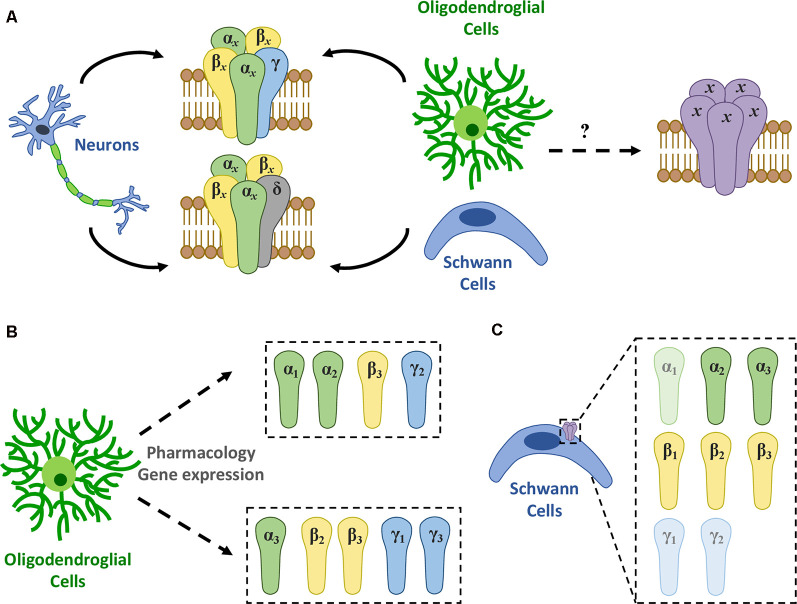
GABA_A_R expression in myelinating cells. **(A)** There are 11 different GABA_A_R subtypes described, mainly heteromeric receptors with α*x*β*x*γ*x* or α*x*β*x*δ (where *x* represents any subtype of a given subunit) stoichiometry (Olsen and Sieghart, 2008). Among them, a single type of GABA_A_R appears to be present in oligodendrocytes though its molecular nature remains elusive. **(B)** Recent evidence using gene expression and pharmacology shows that GABA_A_Rs in oligodendroglia are heterogeneous and may change in subunit composition during differentiation. Thus, at least two novel subtypes were identified: one formed by a combination of α1 or α2, β3, and γ2 subunits (Passlick et al., 2013) and another made up by α3, β2 or β3, and γ1 or γ3 subunits (Arellano et al., 2016). **(C)** In turn, studies using RT-PCR and/or immunohistochemistry conclude that SCs express high levels of α2, α3, β1, β2, and β3, while α1, γ1, and γ2 levels are relatively low in these cells (Magnaghi et al., 2006).

GABA_A_Rs are integral membrane ion channels—permeable to Cl^−^ and HCO_3_^−^ anions—composed of five subunits that mediate the major form of fast inhibitory neurotransmission in the CNS (Olsen and Sieghart, 2008; Doyon et al., 2016). There are, at least, 19 distinct GABA_A_R subunit genes, which include 6 α (α1-α6), 3 β (β1-β3), 3 γ (γ1-γ3), 3 ρ (ρ1-ρ3), and 1 gene of the respective δ, ε, θ, and π subunits (Sieghart and Savić, 2018). This diversity results in different homomeric or heteromeric subunit combinations that may have specific locations in the CNS, particular pharmacology, and, consequently, distinctive functional characteristics (Vogt, 2015). The subunit profile that forms GABA_A_Rs depends on several factors including brain region, cell type, developmental stage, and physiological or pathophysiological conditions (Levitan et al., 1988; Seeburg et al., 1990; Waldvogel and Faull, 2015). Currently, 11 GABA_A_R subtypes with different subunit combinations have been identified, being most of them heteromeric receptors formed by α*x*β*x*γ*x* or α*x*β*x*δ (where *x* represents any subtype of a given subunit; Figure 1A), whereas others are homomeric receptors formed by ρ subunits (Barnard et al., 1998).

### Oligodendroglial Cells

Activation of GABA_A_Rs is relevant for the modulation of myelinating cell physiology (Magnaghi, 2007; Vélez-Fort et al., 2012), however, the specific subunit composition of GABA_A_Rs expressed in these cells remains unknown. Electrophysiological recordings in OPCs and OLs reveal differences between the response of the GABA_A_R expressed in these cells and those expressed in neurons and astrocytes (Table 1), suggesting the presence of a novel GABA_A_R subtype with unique stoichiometry in the oligodendroglial lineage (von Blankenfeld et al., 1991; Williamson et al., 1998; Vélez-Fort et al., 2012; Arellano et al., 2016).

**Table 1:** Pharmacological properties of neuronal and oligodendroglial GABA_A_Rs.

	Neurons		
Drug	Synaptic (α*x*β*x*γ2)	Extrasynaptic (α*x*β*x*δ)	Oligodendroglial cells
**GABA**	Low EC_50_ 1–30 μM	Low EC_50_ 0.5 nM–10 μM	High EC_50_ 70–100 μM
	(Gibbs et al., 1996; Baur and Sigel, 2003; Mortensen et al., 2012)	(Brown et al., 2002; Wallner et al., 2003; Mortensen et al., 2012)	(Williamson et al., 1998; Arellano et al., 2016)
**THIP**	No effect	+	No effect
	(Mortensen et al., 2010)	(Brown et al., 2002; Meera et al., 2011)	(Arellano et al., 2016)
**Zn^2+^**	Low or No effect	−	−
	(Hosie et al., 2003)	(Carver et al., 2016)	(Bronstein et al., 1998; Passlick et al., 2013; Arellano et al., 2016)
**β-CCB**	− or No effect	No effect	+
	(Peña et al., 1986; Cisneros-Mejorado et al., 2020)	(Jiménez-González et al., 2011)	(Arellano et al., 2016; Cisneros-Mejorado et al., 2020)
**DMCM**	−	−	−
	(Peña et al., 1986)	(Brown et al., 2002)	(von Blankenfeld et al., 1991; Arellano et al., 2016)
**Diazepam**	+	No effect	+
	(Walters et al., 2000; Goodkin and Kapur, 2009)	(Goodkin and Kapur, 2009)	(von Blankenfeld et al., 1991; Passlick et al., 2013; Arellano et al., 2016)
**Indiplon**	+	No effect	No effect
	(Petroski et al., 2006)	(Michelsen et al., 2007)	(Arellano et al., 2016)
**Flunitrazepam**	+	No effect	+
	(Goodkin and Kapur, 2009)	(Goodkin and Kapur, 2009)	(von Blankenfeld et al., 1991; Arellano et al., 2016)
**Loreclezole**	+*	+*	+
	(Wingrove et al., 1994)	(Wingrove et al., 1994)	(Arellano et al., 2016)

(+) Potentiator/agonist; (−) Inhibitor; (*) β2 or β3 subunit required.

von Blankenfeld et al. (1991) suggested that GABA_A_Rs in murine OPCs and OLs carry a γ subunit required to form the benzodiazepine binding site, as they observed potentiation of the GABA response in these cells with classic benzodiazepines. Moreover, the inverse agonist β-carboline methyl 4-ethyl-6,7-dimethoxy-9H-β-carboline-3-carboxylate (DMCM) reduced the GABA-induced current responses in oligodendroglial cells, unlike what happens in astrocytes. Contrary to that, Williamson et al. (1998) reported no influence of flunitrazepam or DMCM in the response elicited by GABA in rat-derived OPCs, indicating an absence of the γ subunit in the GABA_A_Rs expressed by these cells. This observation was supported by the inhibitory effect of Zn^2+^, which is characteristic of receptors that lack the γ2 subunit. RT-PCR analyses conducted in the same study did not find expression of γ2, α1, α6, and δ subunit mRNAs in OPCs. Although amplification of other subunits was demonstrated, the results were interpreted with caution since the preparation was 85% pure for OPCs and the presence of GABA_A_Rs from other cell types could not be excluded. In a third study conducted by Bronstein et al. (1998), the GABA response of an immortalized murine glial cell line that expresses mature myelin proteins was insensitive to diazepam and sensitive to Zn^2+^, reinforcing the idea of γ-subunit absence.

Later, Passlick et al. (2013) reported the expression of two types of GABA_A_Rs in hippocampal NG2 cells from juvenile mice by functional and pharmacological analyses and single-cell RT-PCR. NG2 cells from this brain area express, on the one hand, postsynaptic GABA_A_Rs comprised of a combination of α1, α2, β3, γ1, and γ2 subunits and, on the other hand, they have extrasynaptic GABA_A_Rs mostly lacking the γ2 subunit (Figure 1B).

Our studies of GABA_A_R responses conducted in cultured immature OLs from the rat forebrain and mature OLs from the optic nerve showed that these cells are diazepam-sensitive, suggesting once more the presence of a γ subunit (Arellano et al., 2016). This positive modulation by benzodiazepines was observed when using low GABA concentrations (≤EC_30_), which may explain the discrepancies with previous studies. Concerning the specific subtype of γ subunit, two observations indicate that γ2 may not contribute to oligodendroglial GABA_A_Rs: (1) Zn^2+^ blocks GABA responses; and (2) indiplon, a positive allosteric modulator acting on γ2 subunit-containing receptors, does not modulate GABA currents. Therefore, these receptors likely contain either γ1 or γ3 subunit (Arellano et al., 2016).

Regarding β subunits, potentiation of the GABA response by loreclezole suggests the presence of β2 or β3 subunits, since β1 subunit-containing receptors are insensitive to this drug (Arellano et al., 2016). Finally, concerning α subunits, α3 is the most likely candidate because it forms receptors with low sensitivity to GABA (Karim et al., 2013), as it is the case of OLs (EC_50_ between 70 and 100 μM).

Together, these pharmacological studies suggest that the composition of the GABA_A_R expressed in rat-derived oligodendroglial cells is a combination of the α3, β2 or β3, and γ1 or γ3 subunits (Figure 1B). Also, we confirmed the expression of the α3 subunit by immunocytochemistry in cultured OLs (Arellano et al., 2016). These observations are supported by previous functional genomic analyses performed in OLs (Cahoy et al., 2008). Moreover, RNA sequencing (RNA-Seq) transcriptional analyses of purified NG2 cells obtained from P17 mice revealed that the α3 subunit is the most expressed α-subtype, while among β subunits, β3 and β2 are much more abundant than β1. Lastly, γ1 is expressed at much higher levels than γ2 and γ3 (Larson et al., 2016).

**Figure 2:**
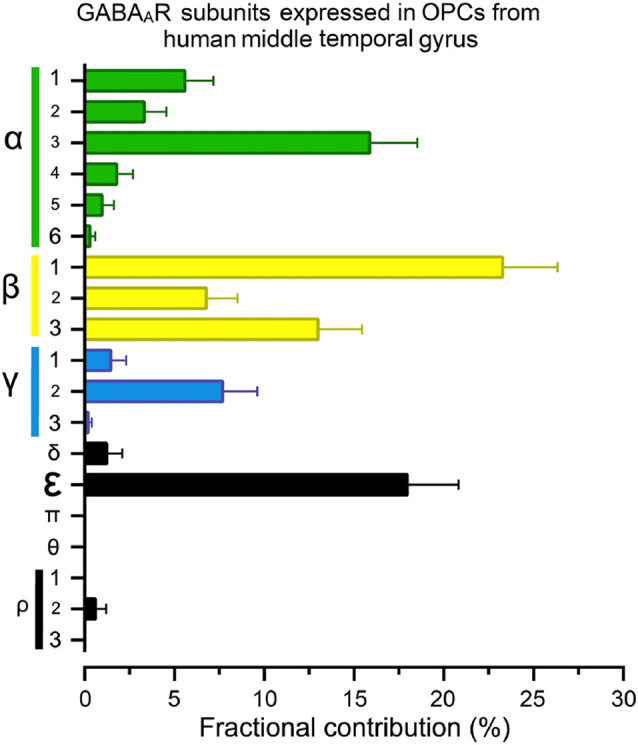
The fractional contribution (FC) of GABA_A_R subunits in human oligodendrocyte progenitor cells (OPCs). Normalized gene expression levels for all 19 GABA_A_R subunits expressed as a percentage (mean ± S.E.M.) of the total available pool of mRNA for GABA_A_Rs in OPCs (PDGFRα^+^ cells) from human brains, estimated from publicly available datasets (Hodge et al., 2019; https://celltypes.brain-map.org/rnaseq). The single-nucleus analysis used normalized RNA-Seq datasets from the middle temporal gyrus isolated from six subjects with no known neuropsychiatry or neuropathological history (three males and three females; 35–66 years old). Gene expression level in each dataset was transformed into FC (Sequeira et al., 2019). FC is defined as the percentage of the expression level of each subunit gene (signaled in the “y” axis) to the sum of the 19 genes for GABA_A_Rs subunits within each human/cell. Detailed demographic characteristics, as well as technical white papers for data processing and quality control, can be downloaded from the same site. Confirmatory analysis of OPC markers enrichment and lack of neuronal markers were performed for all datasets.

An important factor for the diversity of GABA_A_Rs expressed and the subunits involved in their conformation will undoubtedly be the species. For example, regarding humans, a recent study of the GABA_A_R-subunit expression in OPCs isolated from the middle temporal gyrus of healthy adults—based on the single-nucleus RNA-Seq analysis by Hodge et al. (2019)—showed that OPCs from this brain area express high mRNA levels of α3, all β subunits, γ2 and, interestingly, the ε subunit (Figure 2). These mRNAs, if translated and incorporated into functional receptors, would increase the variety of potential configurations and may have important functional and pharmacological consequences (Jones and Henderson, 2007; Bollan et al., 2008; Belujon et al., 2009).

### Schwann Cells

GABA_A_-type receptors are relevant to SC physiology (Magnaghi et al., 2001, 2006). However, their pharmacological and functional properties, as well as their molecular identity, are not clear. GABA_A_R subunit composition in rat-derived cultured SCs includes α2 and α3, as well as the three β subunits, while mRNAs of α1 and γ2 subunits have been found at much lower levels (Magnaghi et al., 2006; Figure 1C). Moreover, the presence of α2, α3, and β3 proteins were confirmed by immunocytochemistry. Alpha-2 and β3 subunits are also expressed in SC-like adult stem cells derived from bone marrow or adipose tissue, as their levels are upregulated following SC differentiation *in vitro* (Faroni et al., 2012). Also, GABA_A_R stimulation with muscimol increases the proliferation rate of SCs, meaning that GABAergic signaling has an important role in these cells (Magnaghi et al., 2006). However, despite these important findings, there is still little knowledge about the specific composition of GABA_A_Rs expressed in differentiated SCs.

### GABA_B_ Receptors

**Figure 3:**
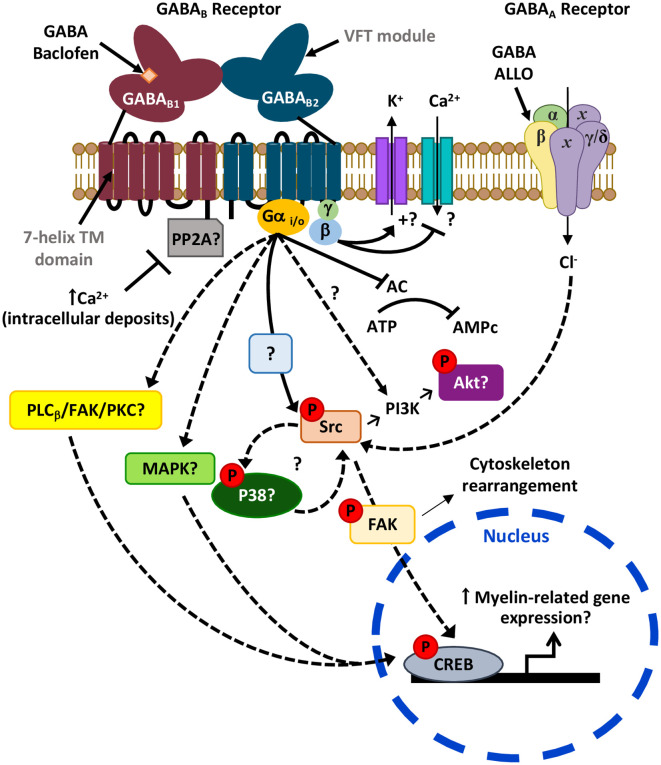
Possible signaling pathways downstream GABARs leading to myelination. Activation of the Gi/o-linked GABA_B_R may reduce CREB phosphorylation as it is negatively coupled to adenylyl cyclase (AC; Luyt et al., 2007). Alternatively, it may also induce CREB phosphorylation possibly *via* activation of PLC_β_/FAK/PKC or MAPK cascades, as observed in neurons (Carlezon et al., 2005; Zhang et al., 2015). Moreover, activation of GABA_B_R leads to Src phosphorylation (Serrano-Regal et al., 2020) that could ultimately induce CREB activation. Phosphorylation of Src may also lead to Akt phosphorylation *via* PI3K as observed earlier (Barati et al., 2015), and contribute to a positive feedback loop with p38MAPK (Mugabe et al., 2010; Lin et al., 2015), which is involved in myelination (Fragoso et al., 2003, 2007). On the other hand, the GABA_B1_ subunit of GABA_B_R might be sequestered by phosphatases like PP2A (as occurs in some neurons), a mechanism that blocks its activity and can be reverted by high intracellular Ca^2+^ levels (Li et al., 2020). Finally, the GABA_A_R allosteric modulator ALLO regulates Schwann cell (SC) myelination *via* Src-FAK signaling, involving cytoskeleton reorganization (Melfi et al., 2017).

GABA_B_Rs are G-protein coupled receptors (GPCRs) responsible for the slower and prolonged GABA-mediated inhibitory transmission. They were first described pharmacologically as bicuculline-insensitive metabotropic receptors that were activated by the GABA analog baclofen (Bowery and Hudson, 1979; Hill and Bowery, 1981). Functional GABA_B_Rs are heterodimers constituted by two receptor subunits, GABA_B1_ and GABA_B2_, that cooperate to perform signal activation (Kaupmann et al., 1998; Kuner et al., 1999). GABA_B1_ is responsible for ligand binding, while GABA_B2_ contains binding sites for allosteric modulators (Galvez et al., 2001; Binet et al., 2004), couples with G_i/o_-protein, and is necessary for trafficking the heterodimer to the cell surface, where the receptor becomes active (Calver et al., 2000; Couve et al., 2000). Among the effector elements involved in GABA_B_R signaling pathways in neurons are voltage-gated Ca^2+^ channels (VGCC), inwardly-rectifying potassium channels (Kir) and adenylyl cyclase (AC; Bowery et al., 2002; Bettler et al., 2004; Figure 3). However, the specific coupling of GABA_B_Rs to the molecular effector may differ depending on the cell type and region analyzed (Booker et al., 2018).

### Oligodendroglial Cells

Myelinating cells express both subunits of GABA_B_Rs, which are negatively-coupled to AC (Magnaghi et al., 2004; Luyt et al., 2007). However, their functional characteristics are not as well known as in the case of neurons. Regarding oligodendroglial cells, we recently confirmed by immunocytochemistry and RT-qPCR the expression of GABA_B1_ and GABA_B2_ subunits in OPCs and OLs from the rat cerebral cortex and in OLs from the optic nerve (Serrano-Regal et al., 2020). We also performed calcium imaging assays and electrophysiological recordings in these cells and observed that baclofen does not modify their response to KCl 50 mM, calcium influx, and Kir currents. These results strongly suggest that GABA_B_Rs in oligodendroglial cells are not coupled to Ca^2+^ and Kir channels in the same manner as in other cell types (Serrano-Regal et al., 2020). Likewise, GABA_B_Rs from CA1 somatostatin interneurons, unlike pyramidal neurons, are not coupled to the canonical Kir3 signaling cascade (Booker et al., 2018), which suggests a functional diversity of downstream effectors depending on cell type and location, and warrants the need of exploring these features in OLs as well as in SCs.

Charles et al. (2003) did not find any colocalization of GABA_B1_ subunit and MBP expression in myelinating OLs in the white matter of the rat spinal cord and suggested that GABA_B_R expression in developing OLs decreases during differentiation. Following this, Luyt et al. (2007) observed downregulation of the GABA_B1_ subunit in mature OLs from the mouse periventricular white matter, while the expression of GABA_B2_ remained constant. As they reported changes in GABA_B1_/GABA_B2_ ratios in mature OLs, they raised the possibility that one subunit alone or in combination with another protein could make GABA_B_R functional in different cell types (Calver et al., 2000; Luyt et al., 2007). In contrast, our recent results show that cultured oligodendroglial cells from the rat forebrain and mature OLs from the optic nerve express GABA_B1_ and GABA_B2_ subunits at different stages of maturation, as well as mature OLs from the juvenile and adult rodent *corpus callosum* *in vivo* (Serrano-Regal et al., 2020). These discrepancies can be explained by the different regions analyzed, as GABA_B1_/GABA_B2_ expression exhibits important regional variations (Luyt et al., 2007). Since oligodendroglial cells are extremely diversified, the different cells targeted in these studies may correspond to distinct oligodendroglial and OPC subpopulations (Marques et al., 2016; Spitzer et al., 2019; Marisca et al., 2020).

### Schwann Cells

SCs also express different isoforms of the GABA_B_R, such as -1a, -1b, -1c, and -2 (Magnaghi et al., 2004, 2006).

Downregulation of GABA_B_R expression occurs in pre- and non-myelinating SCs (Corell et al., 2015). Neurosteroids modulate the expression of GABA_B_R subunits in cultured SCs and, as GABA_B_R is downregulated with age in the PNS, the GABA synthesized in the adult sciatic nerve acts through the ionotropic GABA_A_R, present both in neurons and SCs (Magnaghi et al., 2006). Interestingly, the conditional knockout of the GABA_B1_ subunit in SCs changes the expression of GABA_A_R subunits α3, α4, β1, and δ (Faroni et al., 2019), suggesting that GABA_B_Rs in these cells regulate somehow the expression of GABA_A_Rs and/or their subunits.

Overall, more detailed analyses such as single-cell RNA-seq would help to better figure out the expression of GABA_B1_ and GABA_B2_ subunits along the oligodendroglial and SC lineages.

## Potential GABA Synthesis and Release in Myelinating Cells

Although GABAergic neurons are the main source of GABA (especially in the CNS), GABA synthesis also occurs in glial cells (Seiler et al., 1979; Angulo et al., 2008; Héja et al., 2012). Two potential pathways for GABA synthesis have been described in brain-derived glial cells. GABA is mainly produced through the classical pathway as a result of glutamate decarboxylation by the glutamic acid decarboxylase (GAD) enzymes (Roberts and Frankel, 1950). In neurons, the two isoforms of GAD—GAD_65_ and GAD_67_—differ in their catalytic and kinetic properties and their subcellular distribution (Kaufman et al., 1991). Also, GABA can be synthesized from the monoacetylation of putrescine with the participation of the monoamine oxidase B (MAO_B_) enzyme in the non-classical pathway (Seiler et al., 1973).

Consistent with an RNA-seq transcriptome and splicing database (Zhang et al., 2014), oligodendroglial cells express *gad1*, *gad2*, and *maob* mRNAs. GAD_67_ mRNA (*gad1*) is greatly expressed by OPCs, although its levels decrease notably as they differentiate into mature myelinating OLs. However, *gad2* is expressed to a lesser extent throughout the oligodendroglial lineage. Regarding *maob*, OPCs and myelinating OLs express higher levels than newly formed or immature OLs (Zhang et al., 2014). Accordingly, we confirmed the presence of GAD_65/67_ by immunocytochemistry and western blot in rat-derived cortical oligodendroglial cells at 1, 3, and 6 days *in vitro* (Serrano-Regal et al., 2020). Similarly, we verified the expression of the MAO_B_ enzyme in these cells at the same time points. These results indicate that cultured oligodendroglial cells may synthesize GABA by the two alternative pathways mentioned above. Indeed, we found GABA immunostaining in cortical and optic-nerve derived oligodendroglial cells at different stages of maturation (Serrano-Regal et al., 2020). Possibly, GABA is synthesized by one pathway or another depending on the stage of maturation of the cells, as GABA may have different roles in OPCs vs. mature OLs.

GAD_67_ is present in SCs and its levels increase in the presence of the progesterone metabolite allopregnanolone (ALLO; Magnaghi et al., 2010). Moreover, Corell et al. (2015) demonstrated the presence of GABA and GAD_65/67_ in premyelinating and non-myelinating SCs. These findings show that SCs both produce and store GABA.

Together, these observations indicate that OLs and SCs synthesize and store GABA, which could be released by reversal operation of GABA transporters including GAT-1 and GAT-3, that are expressed by OLs (Fattorini et al., 2017; Serrano-Regal et al., 2020). Also, SCs are capable to take up ambient GABA at concentrations above 1 μM (Brown et al., 1979), though the nature of the transporters involved is still unknown.

## GABA Receptors in OPC/SC Precursor Differentiation and Myelination

In the CNS, OPCs are the main (but not unique) source of remyelination, since they respond to white matter injury, migrate to the lesioned area and differentiate into mature OLs to produce new myelin sheaths (Franklin et al., 1997; Nait-Oumesmar et al., 1999; Hesp et al., 2015). Moreover, surviving mature OLs are also a source of remyelination (Duncan et al., 2018). SCs may also participate, although to a lesser extent, in restoring myelin in the CNS (Zawadzka et al., 2010; García-Díaz and Baron-Van Evercooren, 2020).

Differentiation of OPCs/SCs and myelination are exquisitely coordinated processes mediated by a deep dialogue between neuronal and glial cells that entail the participation of a variety of signals and intercellular communication systems, including ATP, glutamate or GABA neurotransmitter signaling (Li et al., 2013; Faroni et al., 2014; Salzer, 2015; Zonouzi et al., 2015; Arellano et al., 2016; Hamilton et al., 2017; Serrano-Regal et al., 2020), as well as neuronal activity (Wake et al., 2011; Gibson et al., 2014; Fannon et al., 2015). Specifically, GABAergic neurons establish direct synapses with OPCs throughout the CNS, indicating that this communication may control proliferation, migration, differentiation, the establishment of axonal contacts and their wrapping, OPC survival in the adult brain or myelin maintenance (Lin and Bergles, 2004; Kukley et al., 2008; Vélez-Fort et al., 2010; Orduz et al., 2015; Zonouzi et al., 2015; Balia et al., 2017; Mount et al., 2019). However, the precise function of GABA in OPC differentiation and myelination remains controversial.

### GABA_A_ Receptors

Contrary to mature neurons, activation of GABA_A_Rs in OPCs leads to depolarization and an increase in cytosolic Ca^2+^ levels (Kirchhoff and Kettenmann, 1992), resulting from Ca^2+^ influx through activated VGCC (Paez and Lyons, 2020). Thus, a rise in Ca^2+^ in the cytosol may regulate OPC proliferation, migration and maturation and, consequently, OL (re)myelination (Cheli et al., 2016; Santiago-González et al., 2017; Baraban et al., 2018; Krasnow et al., 2018; Marisca et al., 2020).

As oligodendroglial cells constitute a highly dynamic and heterogeneous population, the expression of GABA_A_Rs and their different subunits changes as these cells progress along their lineage, as occurs with the expression of certain ion-channels (Spitzer et al., 2019), and these changes can affect their intercellular relationship and differentiation. In line with this, we observed that oligodendroglial GABA_A_R expression *in vitro* is dependent on the close interaction between axons and OLs, as OLs cultured alone lose GABA responses with differentiation (Arellano et al., 2016). Moreover, the presence of the γ2 subunit, which is associated with a possible role in neuron-OPC synapse formation, decreases with age along with the density of GABAergic synaptic contacts in cortical NG2 cells of mature mice (Balia et al., 2015). Thus, NG2 cells may switch the expression of GABA_A_Rs from synaptic (with γ2 subunit) to extrasynaptic (without γ2 subunit) during development (Vélez-Fort et al., 2010). Surprisingly, genetic inactivation of oligodendroglial γ2 does not affect OPC proliferation and differentiation, while it causes progressive and specific depletion of the OPC pool that lacks γ2-mediated synaptic activity without affecting the oligodendrocyte production (Balia et al., 2017). These observations indicate that GABAergic communication in cortical OPCs through γ2-containing GABA_A_Rs does not play a role in oligodendrogenesis but rather modulates OPC maintenance.

GABAergic signaling regulates OPC population and OPC differentiation and myelination in the cerebellar white matter *in vivo* (Zonouzi et al., 2015). In turn, hypoxia causes a strong downregulation of the GABAergic synaptic input from local interneurons to OPCs (NG2 cells) as well as an increase in the proliferation of these cells and a delay in their maturation, which limits myelination. These effects are mimicked in control animals when blocking GABA_A_Rs with their antagonist bicuculline. However, they are reverted when applying tiagabine, a selective inhibitor of the GABA transporter GAT-1 that increases GABA availability in the extracellular space. Treatment with tiagabine results in a decrease of NG2 cell proliferation and an increase of myelinating OLs, reverting the hypomyelinating effect caused by perinatal hypoxia (Zonouzi et al., 2015). These findings strongly suggest that GABAergic signaling (either neuronal activity-dependent or independent) influences OPC development and differentiation and, therefore, it may help to develop novel therapies to improve OPC differentiation into damaged brain areas.

GABAergic signaling through GABA_A_Rs may also be relevant for the stronger remyelination that occurs following focal demyelination in the *corpus callosum* of late pregnant rats compared to virgin and postpartum ones (Kalakh and Mouihate, 2019). This pregnancy-associated promyelinating effect was lost when either the GABA_A_R was blocked or when 5α-reductase, the rate-limiting enzyme for the endogenous GABA_A_R activator ALLO, was inhibited (Kalakh and Mouihate, 2019). Moreover, N-butyl-β-carboline-3-carboxylate (β-CCB), a selective drug activating preferentially oligodendroglial GABA_A_Rs, promotes remyelination in a model of gliotoxin-induced demyelination in the rat cerebellar caudal peduncle as assessed using magnetic resonance imaging (MRI) together with myelin staining (Cisneros-Mejorado et al., 2020). Together, these results strongly suggest that GABA_A_R-mediated signaling promotes myelination and remyelination in OLs either directly or indirectly. However, at odds with these data, activation of GABA_A_Rs by endogenous GABA in cortical organotypic cultures reduces the number of oligodendroglial cells and myelination whereas enlarges internode length, influencing the velocity of the nerve impulse propagation (Hamilton et al., 2017). These effects may be due to GABA released by glial cells. However, we could not assess this idea as gabazine treatment of OPC cultures had no significant effect on myelin protein production (Serrano-Regal et al., 2020).

Neurosteroid therapy is another pharmacological approach to modulate GABA_A_R activity in the nervous system, as they act as allosteric modulators of these receptors in the nanomolar concentration range (Lambert et al., 2009). Indeed, progestin ALLO increases myelin basic protein (MBP) production in rat-derived cerebellar organotypic slices, an effect that requires GABA_A_Rs (Ghoumari et al., 2003). This observation points to these receptors as mediators of myelination. The effects of neurosteroids are of special interest in postnatal development, as they may help to prevent neurodevelopmental disorders associated with preterm birth (Shaw et al., 2019). ALLO, which is mainly synthesized in the placenta, has an important role during nervous system development. In premature neonates, ALLO concentration decreases abruptly and this decrease is associated, in part, with hypomyelination (Shaw et al., 2015). Consequently, experimental administration of the ALLO analog ganaxolone as replacement therapy in guinea pig-preterm neonates showed positive effects on myelination, through its interaction with GABA_A_Rs. Therefore, neurosteroid replacement could be a good therapeutic option to improve myelination in this condition (Shaw et al., 2019).

Neurosteroids may also enhance GABA_A_R function in SCs. Thus, ALLO acting *via* GABA_A_ receptor can influence peripheral myelin protein 22 (PMP22) synthesis (Magnaghi et al., 2006). Moreover, ALLO modulates SC morphology, motility, and myelination in SC/dorsal root ganglia neuron (DRG) co-cultures *via* the Src/focal adhesion kinase (FAK) pathway, a signaling cascade that involves GABA_A_Rs and relies on actin rearrangements (Melfi et al., 2017). Therefore, neurosteroids represent a promising molecular approach for the treatment of peripheric pathologies. Together, the studies discussed in this section connect GABA_A_R signaling with OPC/SC differentiation and/or myelination using pharmacological approaches. However, the results observed cannot be solely attributed to the action of GABA on GABA_A_Rs. Although pharmacology may be a good strategy to enhance OPC/SC differentiation and myelination in pathological conditions, potential side-effects must also be considered. To minimize them, it would be of great interest to use more specific drugs acting on myelinating cell GABA_A_Rs and/or to use genetic approaches to specifically target the different GABA_A_R subunits expressed in these cells.

### GABA_B_ Receptors

An early study suggests that GABA_B_Rs may be relevant for OPC development as baclofen increases migration and proliferation in cultured OPCs derived from periventricular white matter (Luyt et al., 2007). However, more recent studies could not confirm those findings as baclofen did not change OPC proliferation or total OPC number in dissociated and organotypic cultures derived from the cortex (Hamilton et al., 2017; Serrano-Regal et al., 2020). This discrepancy could reflect the different brain areas studied. Thus, different subpopulations of OPCs may exist in white and gray matter that behave differently or have different responses to baclofen, as proposed by Luyt et al. (2007). In contrast, baclofen reduced cell proliferation of SC cultures (Magnaghi et al., 2004). However, in dissociated developing DRG primary cultures, in which SCs proliferate spontaneously *in vitro*, baclofen did not affect (Corell et al., 2015).

On the other hand, GABA and baclofen modulate OPC differentiation, as well as the myelination capacity of mature OLs cultured with DRG neurons, pointing out GABA_B_Rs as relevant modulators of OL maturation and myelination (Serrano-Regal et al., 2020). Consistent with these observations, GABA_B_Rs also regulate SC differentiation both in the myelinating and non-myelinating phenotypes. Thus, forskolin-induced SC differentiation *in vitro* correlates with a redistribution of GABA_B1_ and GABA_B2_ subunits of GABA_B_Rs. Indeed, in the cytoskeleton rearrangement that takes place during differentiation, GABA_B_Rs colocalize with f-actin on the SC elongated processes (Procacci et al., 2013).

Apart from being essential for SC commitment to a non-myelinating phenotype during development, GABA_B_Rs are key modulators of neuronal-SC interactions regarding myelination, as GABA_B1_ receptor total null mice showed altered levels of PMP22 and myelin protein zero (P0) as well as thinner myelin sheaths. These mice also presented fiber alterations, which causes changes in pain behavior, gait abnormalities, and motor coordination disturbances (Magnaghi et al., 2008). Together, these findings suggest a role for GABA_B_Rs in the control of SC myelination. Moreover, both GABA_B_R subunits in addition to GABA and GAD_65/67_ were found at the node of Ranvier in a sub-population of myelinated sensory fibers (Corell et al., 2015). Surprisingly, GABA_B_R expression is upregulated in SCs of injured nerves, which may be interpreted as an adaptive response for stimulating the neighboring axons to re-grow distally to the injury (Corell et al., 2015).

Finally, conditional deletion of the GABA_B1_ subunit in SCs altered their proliferation, migration, and myelination capacities, as well as reduced neurite length of co-cultured DRGs (Faroni et al., 2019). Furthermore, molecular and transcriptomic changes were also observed both in SCs and DRGs derived from mice lacking GABA_B1_ subunit in SCs (P0-GABA-B1^fl/fl^). Interestingly, the expression of some GABA_A_R subunits by SCs and DRGs was also altered, indicating a possible role of GABA_B_Rs in regulating the expression of GABA_A_Rs in these cells (Faroni et al., 2019). Similar studies using conditional deletion of GABA_B_R subunits in oligodendroglia will help to understand the role of these receptors and their impact on myelination and pain and motor behavior.

## Possible Signaling Pathways Downstream Gabars Related to Myelination

Myelination, either by OLs or SCs, involves the participation of several intracellular signaling pathways. Indeed, some of those pathways are common in the CNS and PNS. For instance, binding of neuregulins (NRGs) to ErbB receptors activates a sequence of canonical intracellular pathways downstream from many receptor tyrosine kinases (RTKs), such as phosphatidylinositol-3-kinase (PI3K)/Akt/mammalian target of rapamycin (mTOR) or mitogen-activated protein kinases (MAPK; Newbern and Birchmeier, 2010).

Intracellular 3′,5′-cyclic adenosine monophosphate (cAMP) induces cell differentiation and myelination requiring the participation of the cAMP response element-binding protein (CREB). CREB mediates the stimulation of MBP expression by cAMP in OLs (Afshari et al., 2001). Moreover, in mouse-derived cultured SCs, the combined action of cAMP/NRG1 increases the expression of myelin proteins Krox-20 and P0, through a mechanism that relies on the activity of transcription factors from the CREB family (Arthur-Farraj et al., 2011).

The Src family kinases (SFKs) are nonreceptor tyrosine kinases that integrate external signals from both integrin and growth factor receptors and transduce signals related to OL and SC development and myelination (Colognato et al., 2004; Melfi et al., 2017). In particular, signaling pathways downstream the Src-family member Fyn regulate morphological differentiation of OLs, the recruitment of cytoskeleton components, and local translation of MBP (see White and Krämer-Albers, 2014; Quintela-López et al., 2019). GABA_B_R specific activation with baclofen induces Akt phosphorylation, which is dependent on PI3K and Src kinases, promoting chemotaxis and cytoskeletal rearrangement in rat basophilic leukemic cells (Barati et al., 2015). Accordingly, we found that Src-family kinases inhibition abrogates GABA_B_R-induced OL differentiation (Serrano-Regal et al., 2020). This observation corroborates the role of the Src family in OL differentiation through a mechanism dependent on GABA_B_R activation (Figure 3).

GABA_A_Rs are also linked to Src and FAK signaling. The modulation of SC development and myelination by the neurosteroid ALLO in SC/DRG co-cultures occurs *via* Src and FAK signaling activation, which depends on GABA_A_Rs and actin reorganization (Melfi et al., 2017). Besides, Src-family members can interact reciprocally with kinases from the MAPK family, like the serine/threonine-protein kinase p38 MAPK, as c-Src elicits p38 MAPK phosphorylation and the opposite (Mugabe et al., 2010; Lin et al., 2015; Wu et al., 2015). Therefore, it would be of great interest to investigate this kind of interactions in myelinating cells, as p38 MAPK is a key element in the initial steps of myelination in SCs (Fragoso et al., 2003), as well as in OL maturation and myelination since specific p38 inhibitors block *in vitro* myelination of DRGs by OLs (Fragoso et al., 2007). Also, conditional knockout of p38 in oligodendroglial cells leads to defects in myelination early in development (Chung et al., 2015). At odds with those findings, deletion of p38 in the same mouse model increases remyelination after cuprizone-induced demyelination (Chung et al., 2015), while selective deletion of p38α MAPK in OLs did not compromise myelination in a mouse model of periventricular leukomalacia (PVL; Chung et al., 2018). These conflicting pieces of evidence indicate that the precise role of p38 MAPK in SC/OL differentiation and myelination and its relation with GABARs remains to be elucidated (Figure 3).

Since GABA_B_Rs couple negatively to AC in OLs (Luyt et al., 2007), activation of CREB downstream these receptors is not expected due to decreased cAMP levels. However, other intracellular signaling cascades activated downstream G-protein coupled receptors, such as MAPK cascades, may phosphorylate CREB (see Carlezon et al., 2005). Thus, GABA_B_R stimulation in cultured mouse cerebellar granule neurons with baclofen activates CREB *via* PLCβ/FAK/PKC (Zhang et al., 2015). Further clarification of the link between GABA_B_R activation and CREB specifically in myelinating cells is a matter of ongoing study and could contribute to a better understanding of the signaling routes that control myelination and remyelination (Figure 3).

Finally, GABA_B_R function may be modulated by its direct association to protein phosphatase 2A (PP2A), as observed in GABAergic neurons from the rodent ventral tegmental area (Li et al., 2020). Therefore, PP2A-GABA_B_R interaction results in an increase of GABA_B_R dephosphorylation and its subsequent internalization, an effect reverted with high intracellular Ca^2+^ levels. Again, it is worth exploring if these mechanisms also occur in myelinating cells and whether they are relevant to myelin pathology (Figure 3).

## Discussion

GABA is among the signals that drive OLs and SCs to axon interactions. The fact that GABA acts mainly on two different types of receptors—ionotropic GABA_A_Rs and metabotropic GABA_B_Rs—makes it difficult to understand the role of this neurotransmitter in myelinating cell physiology. Moreover, OLs and SCs are highly dynamic cell lineages with different stages of maturation.

While the molecular composition of both GABARs and their mechanisms of action are well described in neurons, their properties in myelinating glial cells remain elusive. Native GABA_A_Rs are composed of multiple subunit combinations with diverse pharmacology, both of which vary regionally, adding a huge heterogeneity to their properties and functions. This diversity is also reflected somehow in GABA_A_Rs in OLs and SCs. Thus, SCs express extra-synaptic subunits (in particular the δ subunit, which is key for neurosteroid affinity), while OLs express subunits commonly found at postsynaptic densities, meaning that GABA_A_Rs of OLs and SCs have different subunit composition and, consequently, different pharmacological profiles and functional behaviors (Faroni et al., 2019). Also, there is a switch in the expression of synaptic to extrasynaptic GABA_A_Rs as OPCs progress in the lineage (Vélez-Fort et al., 2010; Balia et al., 2017).

**Figure 4:**
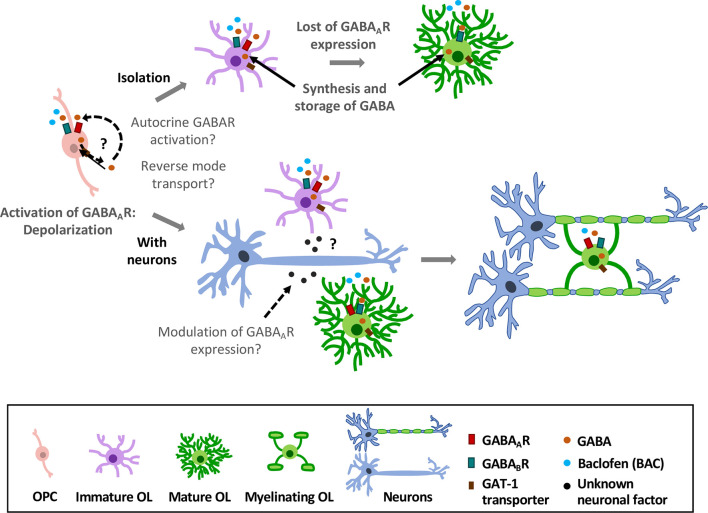
Effects of GABAergic signaling in oligodendroglial differentiation and myelination. OPCs express GABA_A_ and GABA_B_Rs. Activation of GABA_A_Rs causes depolarization in these cells (Kirchhoff and Kettenmann, 1992; Baraban et al., 2018). In absence of axons (top), they lose GABA_A_R expression as they differentiate into mature OLs (Berger et al., 1992; Arellano et al., 2016); however, expression of GABA_B_Rs is largely stable over time (Serrano-Regal et al., 2020). In the presence of axons (low), GABA_A_R expression is modulated by neurons, as OPCs maintain their expression towards the myelinating stage (Arellano et al., 2016). Moreover, oligodendroglial cells express GAT-1 transporter and synthesize GABA, which may be released by reverse GAT-1 operation and activate GABA receptors (GABARs) in an autocrine manner. Exogenous GABA or baclofen promotes oligodendroglial differentiation and myelination *in vitro* (Serrano-Regal et al., 2020).

In oligodendroglial cells, GABA_A_R expression goes down as they mature and acquire a myelinating phenotype (Berger et al., 1992; Arellano et al., 2016). In contrast, GABA_B_R expression is quite stable at all stages (Serrano-Regal et al., 2020). Sustained GABA_A_R expression in oligodendroglial cells depends on the presence of axons, though the mechanisms driving GABA_A_R stabilization remain still unknown. Thus, it is likely that molecules released from neurons in an activity-independent manner may drive GABA_A_R expression (Arellano et al., 2016; Hamilton et al., 2017; Figure 4).

Also, OLs and SCs can synthesize and store GABA, and to take it up from the extracellular fluid through specific GABA transporters (Fattorini et al., 2017). It is therefore conceivable that these cells may release GABA by mechanisms including reverse functioning of the transporters, as observed in other glial cell types (Barakat and Bordey, 2002). Early experiments demonstrated that cultured satellite glial cells from DRG can release [^3^H]GABA in response to a depolarizing stimulus (Minchin and Iversen, 1974). Thus, GABA released by myelinating cells might act in a paracrine or autocrine way (as suggested by Magnaghi, 2007) to, ultimately, modulate their differentiation and/or their myelination capacity (Figure 4). Interestingly, this mechanism operates for instance in polysialylated forms of neural cell-adhesion molecule (PSA-NCAM) progenitor cells in the CNS, which eventually differentiate into glial cells. Thus, autocrine/paracrine loops involving neurosteroids and GABA signaling in these progenitors modulate their proliferation and differentiation (Gago et al., 2004). Synthesis of neurosteroids occurs in SCs (Chan et al., 2000), and may stimulate GABA synthesis in these cells *via* a rapid protein kinase A (PKA)-dependent autocrine loop (Magnaghi et al., 2010). In this way, neurosteroids provide the specific ligand for GABA_A_R activation (Magnaghi et al., 2010). As neurosteroids are involved in promoting SC differentiation and myelination acting through GABA_A_Rs, a possible paracrine/autocrine mechanism could underlie these processes.

GABA_A_ and GABA_B_ receptors may exert opposite roles on myelinating cells, as proposed for SCs in pathological conditions (Faroni and Magnaghi, 2011). Both central and peripheral myelinating cells express GABA_A_ and GABA_B_ receptors, however, this expression depends on the presence of surrounding axons and, as occurs with other receptors and transporters, may vary along the lineage or even depending on the nervous system area (Marques et al., 2016; Spitzer et al., 2019). Thus, depending on the developmental stage of these cells and the GABAR involved, the neurotransmitter GABA may play different physiological functions. Moreover, GABAergic signaling could potentially regulate specific subgroups of cells from the OL/SC lineages in different ways, either action-potential dependently or independently. Thus, it appears that in OPCs GABA acts mostly through GABA_A_Rs to carry out some important functions at the progenitor level (as regulation of the population size, OPC maintenance, axon-glia recognition, differentiation or myelination initiation; Zonouzi et al., 2015; Arellano et al., 2016; Balia et al., 2017; Hamilton et al., 2017; Marisca et al., 2020).

Finally, GABA_B_Rs may contribute to myelin maintenance (Figure 4). In this regard, GABARs may parallel somehow the various functions played by glutamate receptors, as AMPARs are crucial for the early stages of remyelination while NMDARs are relevant for myelin maintenance and to fuel axonal function (Lundgaard et al., 2013; Gautier et al., 2015; Saab et al., 2016).

In sum, understanding the contribution of the GABAergic signaling to OL and SC physiology may be critical to find therapeutic tools to improve remyelination in demyelinating diseases. Meanwhile, it is necessary to clarify in detail the role of GABA in OL and SC differentiation and myelination, and the mechanisms that mediate these responses. To that aim, it will be important to specifically target GABA_A_Rs and GABA_B_Rs either at the progenitor stage or the more mature stages of the myelinating cells. Drugs preferentially acting on GABARs in OLs and SCs will certainly help to successfully tackle these tasks.

## Author Contributions

MS-R and RA wrote the manuscript, designed its content, and prepared the figures and table. LB-C, RO, and AL wrote the manuscript and prepared the figures and table. EG wrote the manuscript. CM and MS-G wrote the manuscript and designed its content.

## Conflict of Interest

The authors declare that the research was conducted in the absence of any commercial or financial relationships that could be construed as a potential conflict of interest.
